# Statistical model of the dynamics of suicides in ukraine before a full-scale war

**DOI:** 10.1192/j.eurpsy.2024.1648

**Published:** 2024-08-27

**Authors:** O. Khaustova, V. Omelyanovich

**Affiliations:** Bogomolets National Medical University, Kyiv, Ukraine

## Abstract

**Introduction:**

The problem of suicides is one of the most critical problems of the public health care system. In Ukraine, official data on the number of deaths and their causes were released by the State Statistics Service only in 2021, on the eve of a full-scale military invasion. This made it possible to conduct statistical analysis and build a mathematical model of the seasonal dynamics of suicidal activity in Ukraine.

**Objectives:**

Develop a statistical model of the dynamics of the number of completed suicides, considering regions of Ukraine and months. For this, a time series of the number of suicides from 2005 to 2021 was created, a mathematical and statistical analysis of the dynamic characteristics of the time series was carried out, and a forecast of the dynamics of the number of completed suicides was built.

**Methods:**

Time series analysis using autocorrelation analysis with the calculation of Leung-Box statistics and the method of seasonal exponential smoothing were applied.

**Results:**

Autocorrelation of the absolute indicators of the number of completed suicides made it possible to construct correlograms for each separate region of Ukraine. In order to ensure the statistical reliability of the autocorrelation coefficients, the number of lags was equal to 50, based on the fact that k ≤ n/4, where k is the maximum number of lags, and n is the number of observations. The correlograms of the regions that characterized the built statistical model of the dynamics of changes in the number of completed suicides were clustered in the form of four groups. The calculation of the coefficient of determination indicated that a high proportion of the total variation for Ukraine as a whole (R2=0.656) and for its individual regions (R2=0.731±0.051) can be explained using the model we built, and the model itself should be evaluated as consistent. Based on the developed model, it was established that the period from March to May, July and, to a lesser extent, January is characterized by the highest number of suicides.

**Conclusions:**

The constructed statistical model of the dynamics of suicides in Ukraine is coherent and statistically reliable. It can be used for forecasting, provided corrections are made, taking into account the social changes of wartime. The study of chronobiological aspects that drew attention during the analysis is promising for further targeted scientific research and may be of practical interest for the creation of national suicide prevention programs in Ukraine.

**Disclosure of Interest:**

None Declared

